# Thromboembolic events in giant cell arteritis: a scoping review

**DOI:** 10.1097/MS9.0000000000005046

**Published:** 2026-04-23

**Authors:** Alexandre Tayler-Gomez, Isabelle Chagnon, Nathalie Routhier, Farah Zarka, Stéphanie Ducharme-Bénard, Jean-Paul Makhzoum

**Affiliations:** a Faculty of Medicine, University of Montreal, Montreal, QC, Canada; b Department of Medicine, Thrombosis Clinic, Hopital Sacre-Coeur, University of Montreal, Montreal, QC, Canada; c Department of Medicine, Vasculitis Clinic, Hopital Sacre-Coeur, University of Montreal, Montreal, QC, Canada

**Keywords:** deep vein thrombosis, giant cell arteritis, pulmonary embolism, risk prediction, venous thromboembolism

## Abstract

**Background:**

Giant cell arteritis (GCA) is a granulomatous vasculitis that can cause severe ischemic complications. GCA also faces an increased risk of venous thromboembolism (VTE), including deep vein thrombosis and pulmonary embolism (PE). Understanding the burden and predictors of VTE in GCA is crucial to improving patient care.

**Materials and methods:**

We conducted a scoping review using PubMed. We identified studies published since August 1990 that reported VTE in adults with GCA. Eligible designs included randomized trials, cohort studies, case-control studies, and case series with ≥5 patients. Data extraction included study characteristics, patient demographics, and VTE outcomes. Findings were synthesized qualitatively, given expected heterogeneity.

**Results:**

Out of 260 records screened, eight studies comprising 36 932 patients with GCA were included. Across studies, 1579 patients (4.3%) experienced VTE. Meta-analyses and large cohort studies consistently reported an elevated risk of VTE compared with controls, with odds ratios ranging from 1.9 to 4.1. The risk was greatest in the first year after diagnosis (relative risks up to 11), declining over time but remaining persistently elevated for at least 5 years. Associated characteristics included inpatient diagnosis, higher comorbidity burden, prior hospitalizations, thrombocytosis, and prior history of VTE, while traditional cardiovascular risk factors were not consistently associated. Mortality was higher in patients with GCA who developed VTE compared to those without.

**Conclusions:**

This review confirms that GCA confers a substantially increased risk of VTE, particularly within the first year after diagnosis. Larger prospective studies are needed to refine prediction models and guide preventive strategies.

## Introduction

Giant cell arteritis (GCA) is a chronic granulomatous vasculitis that predominantly affects individuals over the age of 50 and targets large and medium-sized arteries, with a particular predilection for the extracranial branches of the carotid artery, including the temporal arteries^[^1,2^]^. Clinically, GCA is characterized by a constellation of symptoms including new-onset headache, scalp tenderness, jaw claudication, visual disturbances, and systemic features such as fever and weight loss[2]. If left untreated, GCA can lead to severe ischemic complications, most notably irreversible vision loss, cerebrovascular accidents, and aortic aneurysms or dissections^[^3,4^]^. While the primary clinical focus has historically been on these arterial complications, recent evidence has drawn attention to a potentially increased risk of venous thromboembolic events (VTE), including deep vein thrombosis (DVT), and pulmonary embolism (PE), in patients with GCA[5].

HIGHLIGHTS

- Patients with giant cell arteritis have an increased risk of venous thromboembolism, particularly within the first year after diagnosis, suggesting a temporal link between inflammation and thrombosis.
- Clinical factors such as inpatient diagnosis, higher comorbidity burden, and thrombocytosis appear to contribute to this elevated risk.
- Further prospective and mechanistic studies are needed to elucidate the underlying pathways and inform targeted preventive strategies.

The biological plausibility of this association lies in the profound systemic inflammation that characterizes GCA, which may promote a prothrombotic state. Cytokines such as interleukin-6 and tumor necrosis factor-alpha, which are elevated in active GCA, can induce endothelial activation and dysregulation of coagulation pathways, tipping the hemostatic balance toward thrombosis^[^6,7^]^. This inflammatory milieu may result in increased expression of tissue factor, reduced levels of natural anticoagulants such as protein C and antithrombin, and impaired fibrinolysis[8]. Furthermore, endothelial dysfunction driven by immune-mediated vascular injury can further propagate thrombus formation[9].

Compounding this risk, the standard treatment for GCA involves high-dose systemic glucocorticoids, particularly during the early phase of disease. Corticosteroids are known to influence coagulation by increasing levels of clotting factors and decreasing fibrinolytic activity^[^10,11^]^. In addition, GCA patients, who are often elderly and acutely unwell, may experience prolonged periods of reduced mobility, further contributing to venous stasis and the risk of thromboembolism[12].

Several observational and population-based cohort studies have investigated this association, although findings have been somewhat inconsistent. Some studies report a significantly increased incidence of VTE in the first year following GCA diagnosis, suggesting a temporal relationship between disease activity and thrombotic risk^[^5,13^]^. Others have indicated a more persistent elevation in risk beyond the acute phase, implying that ongoing inflammation or treatment effects may play a role[14]. A recent systematic review and meta-analysis confirmed an overall increased risk of VTE in GCA patients, although the absolute risk remains modest[13].

Given the potential for VTE to contribute to morbidity and mortality in an already vulnerable population, understanding the nature and timing of thrombotic risk in GCA has important clinical implications. It may inform the use of thromboprophylaxis, guide risk stratification strategies, and highlight the need for vigilance in both inpatient and outpatient settings[15]. As research continues to elucidate the immunothrombotic mechanisms underlying GCA, it is essential to integrate these insights into comprehensive patient management frameworks.

The objective of this scoping review is to examine the current evidence regarding the risk of VTE events in patients with GCA, explore the underlying mechanisms that may drive this association, and consider the potential implications for prevention, clinical surveillance, and treatment strategies.

## Methods

This scoping review was conducted in accordance with the Preferred Reporting Items for Systematic Reviews and Meta-Analyses – extension for Scoping Review reporting guidelines. Artificial intelligence was not used in the research or manuscript development^[^16^]^. We employed the Population, Exposure, Comparator, and Outcome (PECO) framework to formulate the primary research question: What characteristics are associated with the development of VTE events, including DVT and PE, in adults diagnosed with GCA?

### Eligibility criteria

We included articles examining the association between GCA and VTE, including DVT and PE. Studies were eligible for inclusion if they involved adult patients aged 50 years and older with a confirmed diagnosis of GCA and reported the occurrence of VTE. The included studies had to be randomized controlled trials, cohort studies, case-control studies, or case series with at least five patients.

Studies were excluded if they were case reports or if the patient population included individuals who were already on anticoagulant therapy at the time of the study. Additionally, we excluded studies that involved patients with hereditary thrombophilia or other concomitant rheumatic diseases.

### Information source and search strategy

To conduct this scoping review, we used the PubMed database to identify relevant articles concerning GCA or temporal arteritis and VTE, including DVT and PE. The search incorporated both Medical Subject Headings vocabulary and free-text keywords. For GCA, terms such as “giant cell arteritis” and “temporal arteritis” were used, while for VTE events, we searched for “venous thromboembolism,” “deep vein thrombosis,” and “pulmonary embolism.”

A publication date filter was applied to limit the search to articles published from August 1990 onward, corresponding with the introduction of the first classification criteria for GCA. The search was conducted on 13 March 2025, without language restrictions, ensuring that articles in any language were included. The results were then screened for relevance and subjected to the eligibility criteria defined for this review.

### Selection process

Titles and abstracts were screened by a single reviewer. Full texts of potentially eligible articles were then reviewed for inclusion. Any uncertainties were resolved through discussion or adjudication by a second reviewer. The study selection process was documented using a PRISMA flow diagram.

### Data extraction

A standardized data extraction form was developed and piloted. A single reviewer extracted data on study design, population characteristics, outcomes, and effect estimates [relative risks (RRs), incidence rate ratio (IRR), odds ratios (ORs), hazard ratios (HRs), and 95% confidence intervals (CI)]. Authors were contacted for additional information when necessary.

### Data synthesis

We conducted a qualitative synthesis of the results from the included studies. No quantitative analysis or summary effect estimate was planned a priori, as we anticipated a high degree of heterogeneity in study designs, populations, outcome definitions, and follow-up durations. Furthermore, we expected the overall quality of evidence to be low, limiting the feasibility and appropriateness of meta-analysis. Instead, findings were narratively summarized and interpreted.

## Results

### Study selection and population characteristics

**Figure 1.**
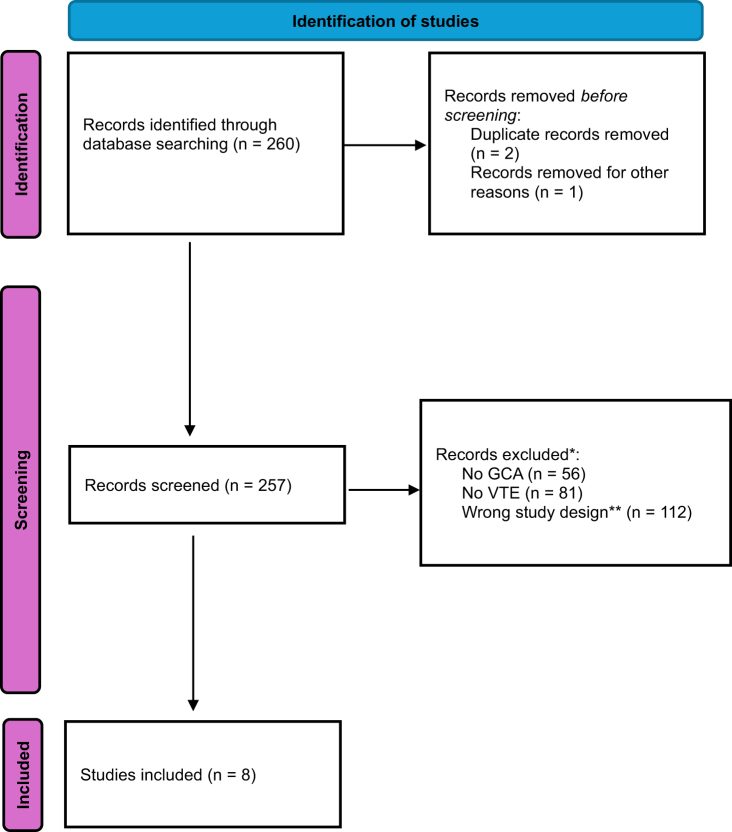
Flow diagram of included studies.

Out of the 260 records screened, 8 studies met the eligibility criteria and were included in our study (Fig. 1). The 8 studies reported on a total of 36 932 patients with GCA, of whom 1579 (4.3%) had VTE. Among studies with available demographic data, 6401 patients (28.4%) were female, and 22 503 (60.9%) had newly diagnosed GCA (Table 1). Only one study directly compared patients with GCA who developed VTE to those who did not; the remaining seven examined patients with both GCA and VTE and described their characteristics.

*Some studies had multiple reasons to be excluded

**For example, case reports, literature reviews, or no information on the characteristics of the patients with VTE.

GCA, giant cell arteritis; VTE, venous thromboembolism.

**Table 1:** Characteristics of studies and of patients included.

	Study design	Total patients with GCA, *n*	Female sex, *n* (%)	Disease subtype (new onset, relapsing, refractory, and unknown)	VTE subtype (DVT and PE), *n* (%)
Amar Muñoz *et al*, 2024^[^17^]^	Retrospective cohort study	188	88 (46.8%)	New onset	2 PE (1.1%)
Aviña-Zubieta *et al*, 2016^[^18^]^	Observational cohort study	909	667 (73.4%)	New onset	18 PE (2%)
					20 DVT (2.2%)
Espinosa *et al*, 2001^[^19^]^	Retrospective cohort study	66	Unknown^a^	Unknown	5 DVT
Ly *et al*, 2017^[^20^]^	Prospective cohort study	428	274 (64%)	New onset	12 PE (2.8%)
					14 DVT (3.3%)
Michailidou *et al*, 2022^[^21^]^	Retrospective cohort study	1535	83 (5.4%)	New onset	28 PE (1.8%)
					61 DVT (4%)
Michailidou *et al*, 2022^[^22^]^	Retrospective cohort study	13 029	798 (6.1%)	New onset	261 PE (2%)
					440 DVT (3.4%)
Rathore *et al*, 2024^[^23^]^	Meta-analysis	14 363^b^	Unknown^c^	Unknown	511 VTE (3.6%)^b^
					133 PE (1.8%)^d^
					Unknown DVT
Unizony *et al*, 2017^[^24^]^	Administrative data analysis	6414	4491 (70%)	New onset	104 PE (1.6%)
					103 DVT (1.6%)

^a^ Among all study participants, 59 (73.8%) were women, a proportion that also includes those with isolated polymyalgia rheumatica. Sex-specific data for the 66 patients with GCA alone were not reported.

^b^ The meta-analysis encompassed 609 954 patients in total, with VTE data available from four studies, including 14 363 patients.

^c^ Although 72.2% of patients in the meta-analysis were female, the four studies evaluating VTE risk did not provide sex-specific data.

^d^ PE data were obtained from three studies, totaling 7567 patients.

### Risk of VTE in GCA compared to controls

A 2024 systematic review and meta-analysis by Rathore *et al* included 14 studies comparing the risk of VTE in patients with GCA versus those without^[^23^]^. GCA was associated with increased odds of VTE (OR = 1.92; 95% CI: 1.73–2.12; *P* < 0.001), DVT (OR = 2.09; 95% CI: 1.50–2.91; *P* < 0.001), and PE (OR = 2.45; 95% CI: 1.38–4.36; *P* < 0.001). No data on patient characteristics were reported.

A 2022 retrospective observational study by Michailidou *et al* compared thromboembolic risk among patients with GCA (*n* = 1535), isolated polymyalgia rheumatica (PMR, *n* = 10 265), overlapping GCA and PMR (*n* = 1203), and osteoarthritis (OA) controls (*n* = 39 009), matched for age and sex^[^21^]^. The IRR for PE was similarly elevated in GCA (3.99; 95% CI: 2.63–5.81) and overlap GCA/PMR (3.82; 95% CI: 2.34–5.88) groups versus OA. For DVT, the IRR was higher in GCA (4.12; 95% CI: 3.13–5.35) than overlap GCA/PMR (1.96; 95% CI: 1.24–2.94). The risk of PE was greatest in the first year after diagnosis (GCA IRR = 11.07; 95% CI: 6.51–18.15; overlap GCA/PMR IRR = 10.20; 95% CI: 5.53–17.71) and declined over time but remained elevated at year 5 (GCA IRR = 3.99; overlap GCA/PMR IRR = 3.82). For DVT, IRRs in GCA decreased from 7.88 in year 1 to 4.12 in year 5, while the overlap GCA/PMR group remained relatively stable (IRR 2.33 in year 1 to 1.96 in year 5).

A 2016 observational cohort by Aviña-Zubieta *et al* (*n* = 909 incident GCA; 9288 matched controls without VTE history) found increased IRRs for VTE (3.58; 95% CI: 2.33–5.34), PE (3.98; 95% CI: 2.22–6.81), and DVT (3.82; 95% CI: 2.21–6.34) in patients with GCA[18]. Stratification by time since diagnosis showed the highest risk in year 1 (VTE IRR = 7.03) with a gradual decline to year 5 (3.69). Similar patterns were seen for PE (IRR 7.23 to 4.09) and DVT (IRR 7.85 to 3.92). HRs were higher in men (HR 4.29; 95% CI: 1.45–12.73) than women (HR 2.19; 95% CI: 1.17–4.10) but similar across age groups.

### Temporal trends in VTE risk

A 2017 cohort study by Unizony *et al* evaluated temporal risk in 6414 new-onset GCA patients and 63 985 matched controls^[^24^]^. VTE risk peaked in the initial months post-diagnosis (RR at 3 months = 9.92; 95% CI: 6.29–15.64) and declined steadily to 2.36 (95% CI: 1.99–2.81) at 96 months, remaining significantly elevated throughout follow-up. The cohort entry point was set 12 months before diagnosis to evaluate pre-diagnostic risk. VTE incidence was 4.2 per 1000 person-years in new-onset GCA versus 2.3 in controls (multivariate-adjusted RR = 1.70; 95% CI: 1.11–2.59). In patients without pre-diagnosis glucocorticoid use (*n* = 3480 GCA; 60 654 controls), incidence rates were 4.0 vs. 2.2 per 1000 person-years, respectively.

In the 2017 inception cohort by Ly *et al* (n = 428 newly diagnosed GCA), 26 patients developed VTE (12 with PE)^[^20^]^. Median time to VTE was 146 days, with most events occurring within the first year.

### Predictors and associated characteristics for VTE

The 2022 cohort study by Michailidou *et al* (13 029 patients with GCA) identified several independent predictors for PE: inpatient diagnosis (HR = 0.65; *P* = 0.03 vs. outpatient), number of hospital admissions in the preceding 5 years (HR = 1.05; *P* = 0.006), and Charlson comorbidity index (HR = 1.13; *P* < 0.001). Predictors for DVT included age (HR = 1.01; *P* = 0.03), Charlson score (HR = 1.10; *P* < 0.001), inpatient diagnosis (HR = 0.67; *P* = 0.01), and prior admissions (HR = 1.04; *P* < 0.001)^[^22^]^. Thrombocytosis (platelets ≥450 × 10^9^/L) predicted DVT in univariable analysis (HR = 3.09; *P* < 0.001). No significant associations were found for smoking, BMI, sex, hypertension, or aspirin/diuretic use. The authors developed a nomogram using age, Charlson score, and prior hospitalizations to estimate 1-year PE and DVT probabilities.

Ly *et al* found that prior VTE (15% vs. 1.7%; *P* < 0.001) and varicose veins (7.7% vs. 0.2%; *P* = 0.002) were more common in those who developed VTE^[^20^]^. Mortality was higher in the VTE group (58% vs. 33%; *P* = 0.01). Baseline labs and symptoms did not differ significantly.

### Risk of thrombosis and auto-antibodies

A 2001 retrospective study by Espinosa *et al* (*n* = 80; 36 isolated GCA, 30 overlap GCA/PMR, 14 isolated PMR) evaluated thrombophilic risk factors^[^19^]^. Twelve patients had “GCA-unrelated thrombosis” (stroke, MI, or DVT), including five DVTs; group allocation was not specified. Antiphospholipid antibodies were present in 39% (anticardiolipin in 30%, anti-prothrombin phospholipid in 36%), but no association with thrombosis was found. Limitations included the inclusion of PMR-only patients, arterial outcomes in the definition of thrombosis, and events occurring before GCA diagnosis in some cases.

## Discussion

This scoping review was conducted to synthesize the available evidence on the risk of VTE, specifically DVT and PE, in patients with GCA, and to identify associated clinical, laboratory, and demographic characteristics. Given the aging population and the increasing prevalence of GCA, it is important to better understand the thrombotic profile of this disease to inform risk stratification, surveillance strategies, and potential preventive interventions.

Across the included studies, several key takeaways emerge. First, there is consistent evidence supporting a markedly increased risk of VTE, particularly during the first year following GCA diagnosis. This early peak in risk was observed in large population-based cohorts and inception studies, with some reports demonstrating an RR as high as 11 in the first year, followed by a gradual decrease but persistent elevation over time. Second, certain clinical characteristics appear to modulate VTE risk. Inpatient status at diagnosis, number of hospital admissions prior to GCA diagnosis, higher Charlson comorbidity index, and thrombocytosis have been associated with increased risk of either PE or DVT. In contrast, traditional cardiovascular risk factors such as smoking, BMI, and hypertension did not consistently differ between GCA patients with or without VTE. Although antiphospholipid antibodies were found in a high proportion of patients in one study, no definitive association with thrombotic events was demonstrated, highlighting the need for more mechanistic investigations. Finally, several mechanistic pathways have been proposed, but these should be interpreted as hypotheses rather than findings directly established by this review. The temporal clustering of events early after diagnosis raises the possibility that intense systemic inflammation, endothelial activation, and glucocorticoid exposure may contribute to a transient prothrombotic milieu. Persistent excess risk over time may reflect ongoing vascular remodeling or chronic endothelial dysfunction. However, the available studies were not designed to formally test these mechanisms, and dedicated translational and prospective investigations are required to clarify the biological drivers of thrombosis in GCA.

Our review has several strengths. It systematically collates and analyzes evidence on both the magnitude and predictors of VTE in GCA. By integrating data from large-scale cohort studies, inception cohorts, and biomarker investigations, this review provides a comprehensive overview of current knowledge and remaining gaps. However, there are also important limitations. The heterogeneity of study designs, outcome definitions, and follow-up durations complicates direct comparisons across studies. Many analyses were limited by a low absolute number of VTE events, resulting in wide CIs and limited power to detect modest associations. Furthermore, few studies provided data on biomarkers or longitudinal laboratory values, limiting our understanding of the underlying mechanisms or predictive profiles.

There remains a clear need for additional studies to address these gaps. Larger prospective cohorts with prespecified VTE outcomes, standardized follow-up, and detailed data on inflammation, coagulation, and immune profiles are warranted. Given the low incidence of VTE events in many cohorts, a case-control study nested within a well-characterized prospective cohort may offer an efficient design to identify independent risk factors. The incorporation of biobanking and biomarker analyses could also enhance understanding of immunothrombotic pathways in GCA. Furthermore, a recent bibliometric analysis in medicine identified clinical decision support and risk prediction as rapidly evolving yet under-developed areas. In GCA, validated AI tools may eventually improve VTE risk stratification and surveillance[25]. Ultimately, refining our knowledge of VTE risk in GCA may support the development of personalized monitoring strategies and targeted prevention efforts for this vulnerable population.

Patients with GCA are at increased risk of VTE, particularly in the first year after diagnosis, with several clinical and comorbidity-related factors contributing to this risk. Further research using prospective cohorts and biomarker-informed approaches is needed to improve risk prediction and guide preventive strategies.

## Data Availability

Not applicable.
